# Tolerability of budesonide/formoterol in patients of Kazakhstan rural regions

**DOI:** 10.1186/2045-7022-5-S2-P21

**Published:** 2015-03-23

**Authors:** Tair Nurpeissov, Temyrzhan Nurpeissov, Gulzada Abdushukurova, Aizhan Avdugalieva, Meruert Gazaliyeva, Alima Shagiyeva

**Affiliations:** 1 SRI of Cardiology and Internal Diseases, ALMATY, Kazakhstan; 2 SRI of Cardiology and Internal Diseases, Republican Allergological Center, ALMATY, Kazakhstan

## Background

Despite all our measures, rates of the allergic diseases in Kazakhstan is steadily increasing every year. Drug allergy and in particular allergic reactions to asthma medication is also a growing problem.

The aim of this study was to assess the frequency of budesonide/formoterol adverse effects.

## Methods

Ninety-three patients with moderate to severe uncontrolled asthma from rural outpatient clinics were included in this study. 54 women and 39 men, their age ranged from 19 to 61 years (average 35.6±4.53). None of them has ever used the combination of budesonide and formoterol before. Every subject underwent a 3-month treatment with budesonide/formoterol, daily doses of budesonide ranged from 640 to 960 mcg depending on the severity of the disease. Tolerability of budesonide/formoterol and its adverse effects occurrence were evaluated.

## Results

Only 5.4% of subject group experienced one or another adverse effect and only in one case, it was sufficient reason to stop the treatment. Patient experienced rapid angioedema with upper airway obstruction on the very first day of treatment. It is necessary to notice, that he has severe polyvalent drug allergy and cannot tolerate majority of other anti-asthmatic drugs. Only other adverse effect that occurred during treatment (in 4.3% of cases) is xerostomia (mouth dryness). It was easily eliminated by rinsing the mouth.

## Conclusions

The combination of budesonide and formoterol showed good tolerability with only 5 cases of adverse effects, majority of which (80%) were easily eliminated and didn’t become a reason to stop treatment.

